# Analysis of autophagy in DLBCL reveals subtype-specific differences and the preferential targeting of ULK1 inhibition in GCB-DLBCL provides a rationale as a new therapeutic approach

**DOI:** 10.1038/s41375-024-02147-4

**Published:** 2024-01-23

**Authors:** Harpreet K. Mandhair, Ramin Radpour, Mira Westerhuis, Yara Banz, Magali Humbert, Miroslav Arambasic, Jörn Dengjel, Andrew Davies, Mario P. Tschan, Urban Novak

**Affiliations:** 1 University of Bern, Department of BioMedical Research, Bern, Switzerland; 2 Department of Medical Oncology, Inselspital, Bern University Hospital, University of Bern, Bern, Switzerland; 3 Graduate School of Cellular and Biomedical Sciences, University of Bern, Bern, Switzerland; 4 Institute of Tissue Medicine and Pathology, University of Bern, Bern, Switzerland; 5 Department of Biology, University of Fribourg, Fribourg, Switzerland; 6 Southampton NHIR/Cancer Research UK, Experimental Cancer Medicines Centre, School of Cancer Sciences, Faculty of Medicine, University of Southampton, Southampton General Hospital, Southampton, UK

**Keywords:** Cancer, Cell biology

## TO THE EDITOR:

Diffuse large B-cell lymphomas (DLBCL) represent approximately 30% of all non-Hodgkin lymphomas [1]. The recognised cell-of-origin (COO) has classified two major DLBCL subtypes: Germinal B-cell (GCB) and Activated B-cell (ABC) lymphomas. Between 20-50% of patients experience relapse and refractory disease following first-line therapy R-CHOP [2]. The recognised molecular heterogeneity contributes to varied clinical outcomes of patients following chemotherapy and targeted agents including ibrutinib, a Bruton tyrosine kinase (BTK) inhibitor [2, 3].

Ibrutinib preferentially targets ABC-DLBCL exhibiting reliance on chronic B-cell receptor (BCR) engagement and hyper NF-κB signalling [3]. Phase I/II trial elucidated that BTK-activity is driven through BCR-dependent signalling with concomitant *MYD88*^*L265P*^ mutations [3]. In contrast, GCB-DLBCL exerts BCR “tonic” activity that is independent of antigen and NF-κB signalling [4, 5]. COO DLBCL subtypes exhibit dependence on phosphatidylinositol-3-kinase (PI3K)/protein kinase-B (AKT) leading to mammalian target of rapamycin complex-1 (mTORC1) activity [5].

Macroautophagy (herein autophagy) maintains cellular homeostasis through the degradation of damaged organelles [6]. Suppression of PI3K-AKT-mTORC1 initiates autophagy through the phosphorylation of unc-51-like kinase-1 (ULK1) [6, 7]. The ULK1 kinase and its complex activation is critical for triggering the autophagic cascade and recruiting microtubule-associated protein 1 light chain-3 (LC3) for the formation of the autophagosome, a double-membraned vesicle [8]. Atypical autophagy facilitates metabolic adaptation in cancer cells [7]. Lysosomotropic autophagy inhibitors including hydroxychloroquine lack potency and efficacy in clinical trials [9]. ULK1 is a druggable serine/threonine kinase [7].

**Fig. 1:**
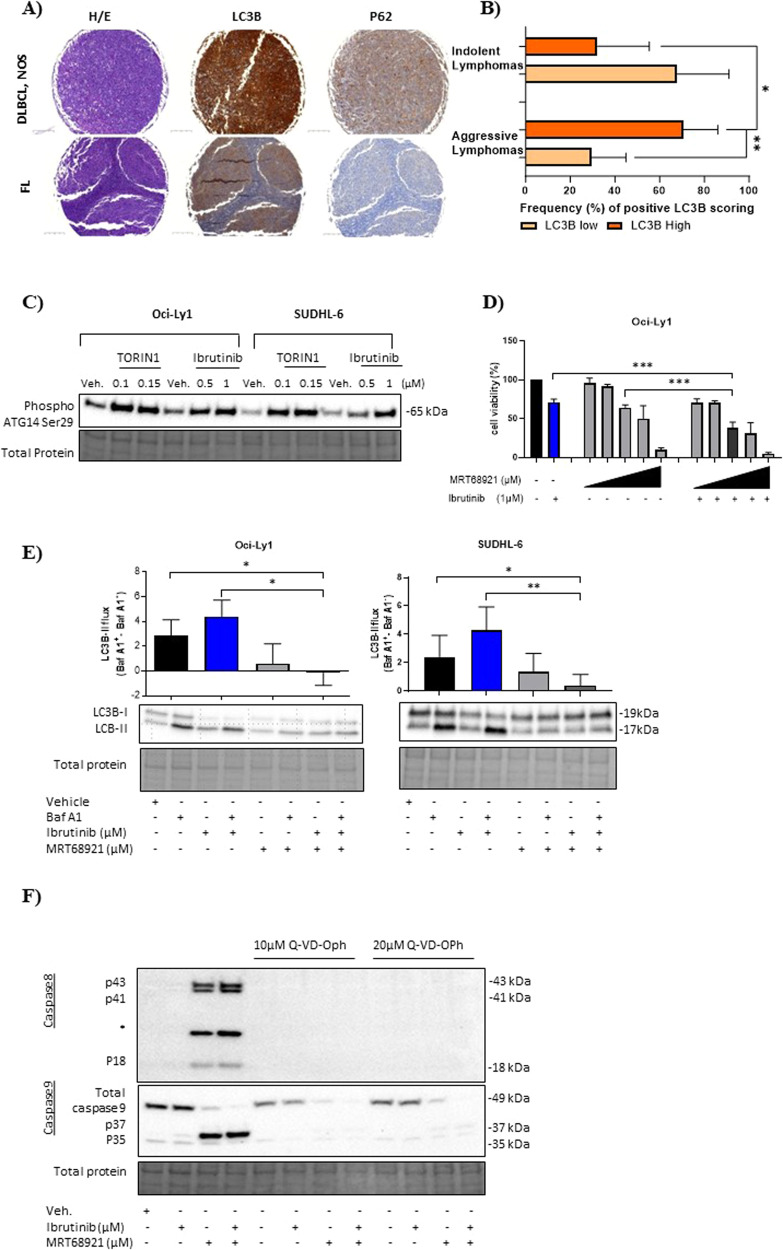
Characterisation of autophagy dependency in primary lymphomas and its preferential targeting in GCB-DLBCL cell lines. **A** Representative images of immunohistochemistry (IHC) staining of excised lymph nodes from aggressive diffuse large B-cell lymphoma (DLBCL, NOS) (top) and follicular lymphoma (FL) (bottom) (10X). Upper and lower lymphoma panel view of autophagy markers: LC3B and p62, and haematoxylin and eosin (H&E) staining. Left panel view of haematoxylin and eosin (H&E) staining, middle and right panels are stained for LC3B and p62. Intense cytoplasmic staining for LC3B and p62 (brown colouration) was defined in DLBCL, NOS compared to FL. **B** 318 primary lymphoma cases were grouped according to their clinical classifications being indolent (*n* = 149) and aggressive (*n* = 169) lymphomas (Supplementary Fig. 1B defines the classification of lymphoma entities). Bar graph summarising the LC3B staining positivity (%) of indolent and aggressive lymphomas. Unpaired student *t* test two-tailed test was used for statistical analysis. **C** Representative blot of two independent experiments for GCB Oci-Ly1 and SUDHL-6 cells treated with vehicle, TORIN1 (0.1 and 0.15 µM) and ibrutinib (0.5 and 1 µM) for 4 h. **D** Cell viability assessment of GCB Oci-Ly1 was subjected to vehicle, ibrutinib (as indicated), ascending MRT68921 (0.5, 1, 2.5, 3 and 5 µM) concentrations in combination for 24 h. *P* values were calculated using one-way ANOVA and Dunnett’s multiple comparisons test, and student *t* test between variables. **E** GCB Oci-Ly1 and SUDHL-6 was treated with vehicle, ibrutinib, MRT68921 and in combination for 24 h in the presence and absence of bafilomycin A1 (Baf A1, 0.2 µM) in the last 2 h. Statistical significance was determined using Mann-Whitney U test. **F** Representative image of Oci-Ly1 treated with vehicle, ibrutinib (1 µM), MRT68921 (2.5 µM) and combination in presence and absence of pan-caspase inhibitor Q-VD-OPh (10 and 20 µM) for 8 h (two independent experiments). Activation of caspase 8 was determined through the engagement of cleavage p43, p41 and active p18. Caspase 9 was activated through cleavage p37 and p35. In the presence of Q-VD-OPh caspase cleavages were abolished. *Non-specific protein. Representative error bars of mean ± SD, *P < 0.05, **P < 0.01, ***P < 0.001, ****P < 0.0001.

Immunohistochemistry of primary lymphoma entities stained for cytoplasmic-LC3B was significant in 70.6% of aggressive compared to 32.3% of indolent lymphomas (Fig. 1A, B, Supplementary Fig. 1A, B). In contrast, no distinct pattern of cytoplasmic-p62 staining was observed within the TMA (Fig. 1A and Supplementary Fig. 1C). This reveals the increased presence of autophagosomes- a hallmark of autophagy [8]. Evaluation of basal autophagy was assessed using bafilomycin A1 (autophagosome-lysosome inhibitor). ABC and multiple GCB cell lines, except GCB HT, demonstrated elevated LC3B-II-dependent autophagic flux at baseline suggesting lymphoma cells are highly autophagy-dependent (Supplementary Fig. 1D). Therefore, we targeted the autophagy initiation with ULK1 inhibitor MRT68921. All DLBCL cell lines showed similar sensitivity to MRT68921 (Supplementary Fig. 1E).

mTORC1 inhibition is reported to activate ULK1-mediated autophagy by activating ATG14 Ser29 [6]. This autophagy marker is indicative of ULK1 stabilisation on the phagophore and positively regulates the ULK1-VPS34 complexes interaction [6]. TORIN1 (mTORC1 inhibitor) and ibrutinib phosphorylated ATG14 Ser29 in GCB Oci-Ly1 and SUDHL-6 cell lines, confirming the presence of ULK1-dependent autophagy (Fig. 1C).

Ibrutinib did not affect the cellular viability of both ABC and GCB cell lines at 24 h (Supplementary Fig. 2A). Next, GCB cell lines were treated with MRT68921 in the presence and absence of ibrutinib. Dual inhibition of BTK and ULK1 cooperatively decreased the cellular viability in multiple GCB cell lines expressing both high (Oci-ly1, Oci-Ly18 and SUDHL-6) and low (HT) levels of LC3B (Fig. 1D and Supplementary Fig. 2B). To ensure MRT68921 blocked LC3B-II turnover in GCB cell lines expressing high basal autophagy we measured LC3B-autophagic flux. Monotherapy of ibrutinib significantly increased LC3B-autophagic flux by 1.5-fold and 1.9-fold in Oci-Ly1 and SUDHL-6 compared to vehicle. Addition of MRT68921 countered ibrutinib-induced LC3B-II turnover and markedly abrogated the autophagic flux by 4.3-fold and 3.9-fold in Oci-Ly1 and SUDHL-6 (ibrutinib vs. combination, Fig. 1E). Proliferating GCB lymphoma cells significantly decreased in the combination treatments demonstrating that MRT68921 augments ibrutinib toxicity (Supplementary Fig. 1C).

Next, we explored whether the observed growth inhibitory effects were exclusive to ULK1 inhibition or could be recapitulated by blocking autophagy using VPS34 inhibitor VPS34IN1. Furthermore, MRT68921 treatment markedly diminished the clonogenic capacity of GCB lymphoma lines and sustained this effect in presence of ibrutinib revealing an additive effect (Supplementary Fig. 2D). GCB Oci-Ly1 and SUDHL-5 were sensitive to VPS34IN1, as evidenced by cell viability, autophagic flux and cellular proliferation assays (Supplementary Fig. 3A-C). VPS34IN1 coupled with ibrutinib significantly decreased the clonogenic activity of lymphoma cells (Supplementary Fig. 3D), however not to the same extent as ULK1 blockade. Pan-caspase inhibitor Q-VD-OPh rescued MRT68921-treated GCB Oci-Ly1 cells from undergoing caspase 8/9 (Fig. 1F) and effectors 3/7 activity dependent apoptosis, as evidenced by an increase in cell viability at 8 h (Supplementary Fig. 4A). Additionally, caspase 3/7 activity was stimulated in GCB HT, Oci-Ly18 and SUDHL-6 during apoptosis (Supplementary Fig. 4B).

Ibrutinib treatment targeted AKT Ser473 phosphorylation, but not MRT68921, suggesting that AKT and ULK1 are unlikely participants in a positive-feedback loop (Supplementary 5A). To confirm that ibrutinib targeted AKT Ser473 multiple PI3K-associated inhibitors were used to demonstrate the dephosphorylation of PI3K cascade in GCB cell lines (Supplementary Fig. 5B). These datasets underscore the need to inhibit these pro-survival pathways simultaneously.

**Fig. 2:**
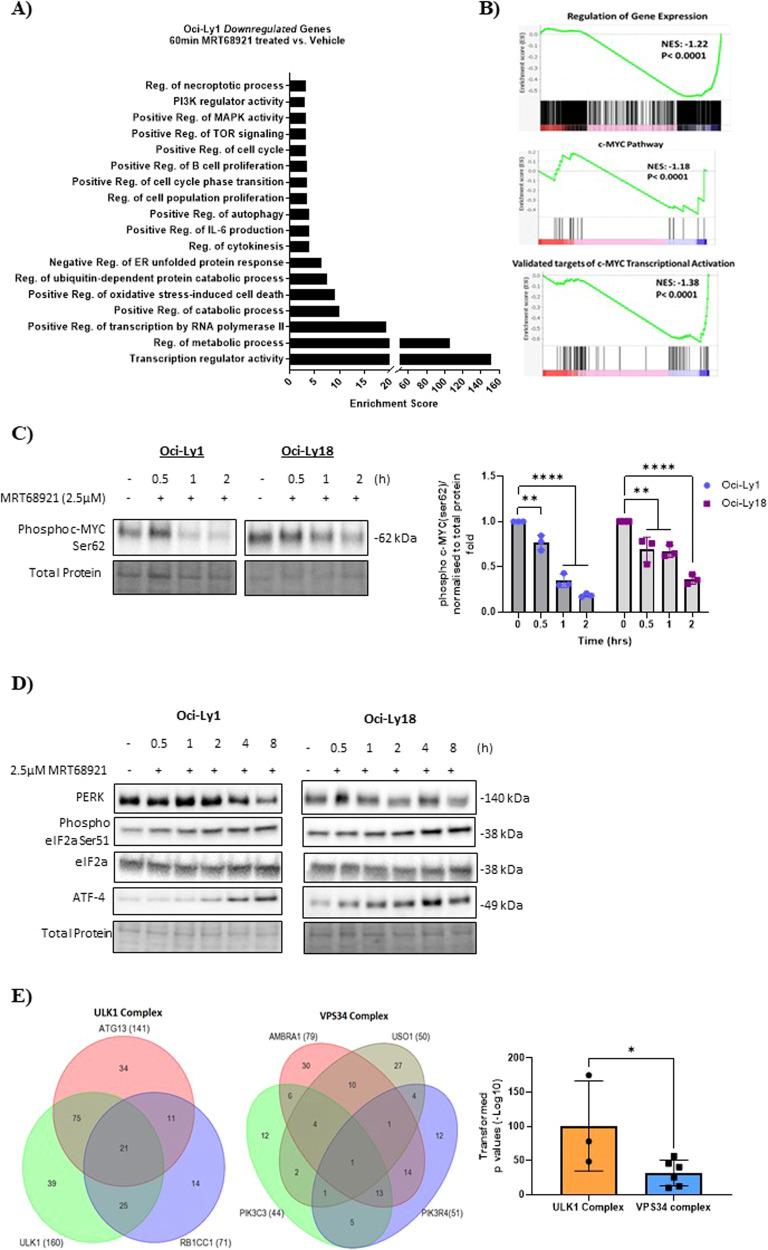
Characterisation of ULK1 inhibition in GCB cell lines and provides a rationale for its targeting in GCB-DLBCL patients. **A** Gene Ontology (GO) analysis of downregulated genes in Oci-Ly1 (MRT68921-treated with 2.5 µM vs. vehicle). **B** GSEA representing negative NES values of genes downregulated associated to regulation of gene expression, c-MYC transcriptional activation and c-MYC pathway. **C** GCB Oci-Ly1 and Oci-Ly18 cell lines treated with MRT68921 for 0.5, 1 and 2 h and phosphorylation of c-MYC Ser62 was measured. This blot represents three independent experiments. The relative protein expression was normalised to the total protein and a fold was generated. One-way ANOVA with post hoc Dunnett’s multiple comparisons test. Representative error bars of mean ± SD, *P < 0.05, **P < 0.01, ***P < 0.001, ****P < 0.0001. **D** Endoplasmic reticulum stress is induced in response to autophagy inhibition. GCB Oci-Ly1 and Oci-Ly18 cells were treated with 2.5 µM MRT68921 in a time-dependent manner. Activation of the PERK pathway was confirmed by phosphorylation of eIF2a and activating transcription factor ATF-4. Immunoblotting was used for detection. **E** GSEA compared autophagy transcripts between gene expression profiling (GEP) of GCB (*n* = 240) and ABC (*n* = 121) patients assigned to R-CHOP treatment arm. Venn diagram illustrating the co-expression of differentially expressed genes from the ULK1 and VPS34 complexes in GCB patients. Increased gene expression of the ULK1 complex is associated with poor overall survival upon R-CHOP treatment. Transformed *p* values from (Supplementary Fig. 9A) were *p*lotted, data presents mean ± SD and student *t* test unpaired two tailed, *P < 0.05.

RNA-sequencing evaluated the differentially expressed genes of GCB Oci-Ly1 and ABC Oci-Ly3 lines conditioned with MRT68921 or vehicle. Heterogeneity between both DLBCL subtypes was assessed using unbiased principal component analysis (Supplementary Fig. 6A). Volcano plots revealed that a total of 359 and 175 genes were differentially expressed in GCB Oci-Ly1 and ABC Oci-Ly3 upon treatment (Supplementary Fig. 6B). Gene ontology (GO) in GCB Oci-Ly1 (MRT68921 vs. vehicle) indicated the downregulation of genes relating to the PI3K-MAPK-TOR signalling, autophagy, and regulation of metabolism. Genes associated to the cell cycle, B-cell proliferation, regulation of IL-6 and oxidative stress-induced cell death were also downregulated (Fig. 2A). Gene enrichment was downregulated in transcripts relating to gene expression, c-MYC pathway and transcriptional activity, as evidenced by gene-set enrichment analysis (GSEA) (Fig. 2B). We validated this result in GCB Oci-Ly1 and Oci-Ly18 cells harbouring c-MYC aberrations [5, 10]. In a time-dependent manner MRT68921 considerably reduced protein levels of c-MYC Ser62 phosphorylation (Fig. 2C), implying that ULK1 post-translationally regulates c-MYC.

Our GO data revealed the activation of the endoplasmic reticulum (ER)-unfolded protein response (UPR). In GCB Oci-Ly1 and Oci-Ly18 we validated this data showing the activation of ER-stress protein PERK-mediated phosphorylation of eIF2α Ser51 in a time-dependent manner (Fig. 2D). The PERK-eIF2α axis is known to attenuate global protein synthesis [11]. Prolonged ER-stress initiates ATF4 to drive its transcriptional activity to restore ER homeostasis or induce apoptosis [11]. In GCB cell lines an increase in ATF4 protein abundance was observed leading to 8 h (Fig. 2D). Subsequently, at 8 h MRT68921 induces apoptosis (Fig. 1F).

MRT68921 treatment in ABC Oci-Ly3 downregulated similar genes to GCB Oci-Ly1 (Fig. 2A and Supplementary Fig. 6C). MRT68921 downregulated genes associated with the proteosome and concurrently an upregulation of gene enrichment in oxidative stress and cell death was detected in ABC Oci-Ly3 (Supplementary Fig. 6C, D). Proteasomal inhibition causes the generation of elevated reactive-oxygen species to induce ER-UPS dependent cell-fatality [12]. Another distinct feature of ULK1 inhibition in ABC Oci-Ly3 is the increased transcripts for NF-κB translational activity (Supplementary Fig. 6C). Moreover, differentially expressed genes associated with the JAK-STAT pathway and inflammatory response was upregulated (Supplementary Fig. 6D). GSEA in ABC Oci-Ly3 confirmed the enrichment of genes relating to acute inflammatory pathway, Il-6 pathway and production compared to GCB Oci-Ly1 (Supplementary Fig. 6E). Assessment of IL-6 secretion indicated that MRT68921 treatment did not impair the high IL-6 levels in ABC cell lines compared to GCB cells (Supplementary Fig. 6F). Also, ABC Oci-Ly3 harbours a c-MYC amplification [5], MRT68921 did not change the c-MYC transcript (Supplementary Fig. 6G). It is purported that JAK1 phosphorylates and activates STAT3 and epigenetically mediates the phosphorylation of chromatin H3Y41-P to regulate *IRF4*, *MYD88* and *MYC* [13]. GAS motifs on STAT3-promoter enables STAT3 to form a positive autoregulatory feedback to regulate the levels of phosphorylated/ unphosphorylated STAT3 [13, 14]. Subgroups of ABC-DLBCL exhibit increased IL-6 signalling through STAT3 and NF-κB activity [14]. c-MYC expression is reported to reduce following *JAK1* silencing in ABC cells [13]. Our in silico analysis based on protein-protein interaction revealed that IL-6 directly interacts with JAK1-STAT3 to regulate c-MYC (Supplementary Fig. 6H). Collectively, these findings highlight the diversity of ULK1-inhibitory pathways in ABC and GCB cell lines.

Gene-expression profiles of primary GCB (*n* = 240) and ABC (*n* = 121) cases treated with R-CHOP from the REMoDL-B trial (ClinicalTrial.gov identifier NCT01324596) [15] was used to assess autophagy-gene *ATG* expression. No difference in autophagy transcripts was detected between the DLBCL COO subtypes. Heatmaps displayed the gene expression of ULK1 and VPS34 complexes in patients (Supplementary Fig. 7A). Kaplan-Meier survival curves identified multiple *ATG*s from the ULK1 complex (*ULK1*, *RB1CC1* and *ATG13*) and VPS34 complex (*AMBRA1*, *PIK3C3*, *PIK3R4* and *USO1*) associated with poor outcome upon R-CHOP treatment (Supplementary Figs 7B, 8). Venn-diagrams were used to compare gene expressions of ULK1 and VPS34 complexes in GCB patients. Patients (co-)expressing *ATGs* were overlapped, and statistical significance was determined. A larger number of GCB patients co-expressed multiple ULK1 complex genes compared to the VPS34 complex (Fig. 2E and Supplementary Fig. 9). The ULK1 complex was significantly upregulated in GCB patients, and this correlated with inferior outcome upon R-CHOP (Fig. 2E and Supplementary Fig. 9).

To our knowledge, the present study is the first to identify that primary aggressive lymphomas and both DLBCL subtype cell lines are substantially autophagy reliant. Our in vitro findings underscore the selective therapeutic benefit of ULK1 inhibition in GCB subtype. Furthermore, the ULK1 complex is significantly upregulated in GCB patients and directly effects treatment response. Our results provide a rationale to target the ULK1 complex in GCB-DLBCL and underscores the importance of using *ATG*s as biomarkers to predict treatment-response.

### Supplementary information

Supplementary Methods

Supplementary Figures

## References

[1] Sehn LH, Salles G (2021). Diffuse large B-cell Lymphoma. N. Engl J Med.

[2] Crump M, Neelapu SS, Farooq U, Van Den Neste E, Kuruvilla J, Westin J (2017). Outcomes in refractory diffuse large B-cell lymphoma: results from the international SCHOLAR-1 study. Blood.

[3] Wilson WH, Young RM, Schmitz R, Yang Y, Pittaluga S, Wright G (2015). Targeting B cell receptor signaling with ibrutinib in diffuse large B cell lymphoma. Nat Med.

[4] Phelan JD, Young RM, Webster DE, Roulland S, Wright GW, Kasbekar M (2018). A multiprotein supercomplex controlling oncogenic signalling in lymphoma. Nature.

[5] Erdmann T, Klener P, Lynch JT, Grau M, Vočková P, Molinsky J (2017). Sensitivity to PI3K and AKT inhibitors is mediated by divergent molecular mechanisms in subtypes of DLBCL. Blood.

[6] Park JM, Jung CH, Seo M, Otto NM, Grunwald D, Kim KH (2016). The ULK1 complex mediates MTORC1 signaling to the autophagy initiation machinery via binding and phosphorylating ATG14. Autophagy.

[7] Ianniciello A, Zarou MM, Rattigan KM, Scott M, Dawson A, Dunn K (2021). ULK1 inhibition promotes oxidative stress-induced differentiation and sensitizes leukemic stem cells to targeted therapy. Sci Transl Med.

[8] Schläfli AM, Berezowska S, Adams O, Langer R, Tschan MP (2015). Reliable LC3 and p62 autophagy marker detection in formalin fixed paraffin embedded human tissue by immunohistochemistry. Eur J Histochem.

[9] Horne GA, Stobo J, Kelly C, Mukhopadhyay A, Latif AL, Dixon-Hughes J (2020). A randomised phase II trial of hydroxychloroquine and imatinib versus imatinib alone for patients with chronic myeloid leukaemia in major cytogenetic response with residual disease. Leukemia.

[10] Mehra S, Messner H, Minden M, Chaganti RS (2002). Molecular cytogenetic characterization of non-Hodgkin lymphoma cell lines. Genes Chromosomes Cancer.

[11] Ron D, Walter P (2007). Signal integration in the endoplasmic reticulum unfolded protein response. Nat Rev Mol Cell Biol.

[12] Pérez-Galán P, Roué G, Villamor N, Montserrat E, Campo E, Colomer D (2006). The proteasome inhibitor bortezomib induces apoptosis in mantle-cell lymphoma through generation of ROS and Noxa activation independent of p53 status. Blood.

[13] Rui L, Drennan AC, Ceribelli M, Zhu F, Wright GW, Huang DW (2016). Epigenetic gene regulation by Janus kinase 1 in diffuse large B-cell lymphoma. Proc Natl Acad Sci USA.

[14] Lam LT, Wright G, Davis RE, Lenz G, Farinha P, Dang L (2008). Cooperative signaling through the signal transducer and activator of transcription 3 and nuclear factor-{kappa}B pathways in subtypes of diffuse large B-cell lymphoma. Blood.

[15] Davies A, Cummin TE, Barrans S, Maishman T, Mamot C, Novak U (2019). Gene-expression profiling of bortezomib added to standard chemoimmunotherapy for diffuse large B-cell lymphoma (REMoDL-B): an open-label, randomised, phase 3 trial. Lancet Oncol.

