# Supplementary material for: Analysis of autophagy in DLBCL reveals subtype-specific differences and the preferential targeting of ULK1 inhibition in GCB-DLBCL provides a rationale as a new therapeutic approach
Source: Leukemia. 2024 Jan 23;38(2):424–9. doi: 10.1038/s41375-024-02147-4 (PMC10844068; doi:10.1038/s41375-024-02147-4)
Supplement: Supplementary file 1 — Supplementary Methods [file 41375_2024_2147_MOESM1_ESM.pdf]

Materials and Methods

Ethical approval

Ethical approval for the use of human specimens was granted by the local ethical committee (Kantonal Ethikkomission Bern, Switzerland, KEK-Nr. 151/14).

Patient tissue samples and Next generation tissue microarray (TMA)

318 primary lymphoma cases with adequate formalin-fixed and paraffin-embedded (FFPE) samples were selected. For each patient sample, small tissue cores of 0.6mm in diameter were punched out of the FFPE tissue blocks using automatic arraying device (TMA Granmaster, 3D Histech, Budapest, Hungary). To simultaneously investigate TMA spots above >350 samples, punches were transferred into a total of three receiver blocks. Samples constructed using next generation TMA (ngTMA)[1] were included in the current study for analysis.

Immunohistochemistry (IHC)

LC3B and p62 staining was performed on the TMA at the Transitional Research Unit of the Institute of Tissue medicine and Pathology in Bern/Switzerland. The immunohistochemistry was performed using an autostainer (Leica BOND RX, Leica Biosystems, Nussloch, Germany). For antigen retrieval, the TMA was pre-treated using a citrate buffer at 100° for 30 min for p62 staining. Pre-treatment for the LC3B staining consisted of tris buffer at 95° for 30 min. The incubation was performed at room temperature. The monoclonal rabbit p62 antibody (LabForce mbl, Nunningen, Switzerland, rabbit polyclonal, #PM0045) at a working concentration of 1:8000 for 120 minutes and the monoclonal rabbit LC3B antibody (Novus Biologicals, Zug, Switzerland, rabbit polyclonal, #NB600-1384) at a working concentration of 1:4000 for 30 min. A detection kit (Bond Polymer Refine Detection, Leica Biosystems) was used for visualisation according to the manufacturer's instructions.

Immunohistochemical scoring and evaluation

Slides were visualized under a microscope to check for staining quality and staining pattern. Cytoplasmic dot-like staining ranging from absent to strong was observed. According to the observations made using the 40x objective magnification, cytoplasmic positivity was assessed semi-quantitatively according to intensity and number of cells with a dot-like staining pattern. The assessment was based on a previously established IHC staining protocol by Schläfli et al, 2015[2]. A score from 0 – 3 was established as follows: score 0, completely negative staining;

score 1, weak dot-like staining; score 2, moderately intense dot-like staining in ≥ 5% of the cells; score 3, intense dot-like staining in ≥ 5% of the cells.

Scoring was performed using "Scorendo" programme, an in-house visual assessment tool for scoring TMA spot images in a randomised and blinded manner. Each TMA spot was analysed by two independent observers (YB, MW) to achieve a concordant score. Slides were scanned with an automatic brightfield scanner (Panoramic 250 FLASH III, 3DHistech, Budapest, Hungry), followed by spot detection. A reference spot for each score (0-3) was used to assure equal and high-quality staining of the lymphoma tissue. Intra- and inter- observer variability was examined.

Population Study using REMoDL-B clinical trial data

Gene expression profiling of REMoDL-B clinical trial (ClinicalTrial.gov identifier: NCT01324596)[3] was obtained. Trial participant data was made available following a Data Sharing Agreement with Southampton Clinical Trials Unit. *In silico* analysis included DLBCL patients from the R-CHOP arm. Transcriptomic data used to examine different gene signatures and influence on overall survival (OS). Gene expression outcome-based cut-points was defined using X-Tile software[4].

Cell Culture

Human ABC DLBCL cell lines HBL-1 and TMD8 were cultured in RPM1 1640 media (Gibco-Life Technologies) supplemented with 1% L-Glutamine (Sigma-Aldrich) and 20% fetal bovine serum (FBS) (Sigma-Aldrich), except U2932 were supplemented with 10% FBS. Oci-Ly3 and RIVA were grown under the same conditions however using Iscove's Modified Dulbecco's medium (IMDM) (SigmaAldrich). GCB DLBCL cell lines Oci-Ly1, Oci-Ly18, SUDHL-5, SUDHL-6, SUDHL-7, and SUDHL10 were also cultured in IMDM, 10% FBS and 1% L-Glutamine. HT and RC-K8 were cultured under similar conditions using RPMI 1640 media. All cell lines were maintained in a humified atmosphere at 37o C and 5% CO2.

HBL-1, TMD8 and U2932 cell lines were gifted by Professor Francesco Bertoni, Institute of Oncology Research, Bellinzona, Switzerland. Professor Riccardo Dalla-Favera, Columbia University, New York, USA, kindly gifted the remaining human DLBCL cell lines.

Compounds

Ibrutinib (S2680, Selleckchemicals), MRT68921 (SML1644-5mg, Sigma-Aldrich), ULK-101 (S8793, Selleckchem), VPS34-IN-1 (B6179, APExBio), Q-VD-OPh Caspase/ Apoptosis Inhibitor (catalogue no. SMPH001, SM Biochemicals), Bafilomycin A1 (Baf A1, LC Laboratories, USA), Everolimus (RAD001) (S1120, Selleckchem), CAL-101 (Idelalisib) (A3005, APExBIO), Capivasertib (AZD5363) (HY-15431, MedChemExpress), Torin 1 (S2827, Selleckchem).

PrestoBlueTM cell viability assay

DLBCL cell lines were seeded at a density of 4x104 cells/ 100µl well in a 96 well plate. Cells were treated with the compound, or vehicle for 24 h. Cell viability was determined using PrestoBlueTM Cell Viability Reagent (A1326, Thermo Scientific). The cells were incubated with 10µL PrestoBlueTM for 30-40 min at 37°C. Absorbance was measured at 570nm with 600nm reference wavelength, using a spector-photometer (Tecan Reader). Cell viability was normalized to vehicle within the same plate. The mean decrease and percentage were calculated.

5-Bromo-2′-deoxyuridine (BrdU) incorporation cell proliferation assay

GCB-DLBCL cell lines were seeded (4x104 / 100µl well) in 100µg/mL ploy-d-lysine (A-003-E, Millipore, USA) treated clear flat bottom 96-well plates. Cells were subjected to the compound compounds, or vehicle in triplicates for 24 h at 37OC. Thereafter, BrdU immuno-assay was conducted following the manufacturer’s instructions. In brief, 10µl of BrdU labelling solution (100µM) was added to each well and the plate was re-incubated for 2 h at 37OC. The labelling solution was removed and 200µl/ well FixDent solution was added and incubated for 30 min at RT. The FixDent solution was removed thoroughly. 100µl/ well anti-BrdU-POD working solution was pipetted into each well and further incubated for 90 min at RT. Anti-BrdU-POD was removed and the plate was washed three times with 200µl/ well PBS. 100µl/ well substrate solution was added to each well and incubated in the dark until a noticeable colour development. The absorbance was measured at 405nm using Tecan plate reader.

Caspase-Glo® 3/7 Assay

2x104 cells/ 100µl well were seeded in a white-walled 96-well plate. Cell lines were treated with their respected conditions for 8 h in triplicates and incubated at 37OC. Caspase-Glo® 3/7 activity was measured using Caspase-Glo® 3/7 Assay (G8093, Promega AG, Switzerland),

according to manufacturer's instructions. Q-VD-OPh was used to inhibit caspase activity at the indicated concentrations. Cells were pre-treated with the inhibitor 1 h prior to the experiment.

Immunoblotting

Whole cell lysates were prepared using urea 8 M urea, 0.5% Triton-X (Sigma-Aldrich), cOmplete protease inhibitor (Roche Diagnostics, Germany) and phosphatase inhibitor (PhosSTOP, SigmaAldrich). Lysed cells denatured in 4x Laemmli sample buffer (Bio-Rad, Switzerland) supplemented with β-Mercaptoethanol (Sigma-Aldrich) at 95OC for 5 min. Lysates were loaded onto 4-20% precast TGX gels (Mini-Protean TGX stain-free gels, Bio-Rad, USA) and transferred to PVDF membranes (Trans-Blot Turbo RTA Transfer Kit, PVDF, Bio-Rad, USA). Membranes were blocked in 5% milk suspended in TBS 0.05% Tween-20 (TBS-T) for 1 h at room temperature (RT). Membranes were incubated overnight with their respective primary antibodies at 4OC overnight. Secondary antibodies were horseradish peroxidase conjugated secondary goat anti-rabbit (7074, Cell Signalling Technology, Switzerland) or goat anti-mouse (7076, Cell Signalling Technology, Switzerland) and detected at 1:3000- 10,000 incubated for 1 h at RT. Membranes were subjected to chemiluminescence (Clarity Western ECL substrate Bio-Rad, USA) for visualisation on the ChemiDocTM MP system (Bio-Rad, Switzerland) and ImageLab software. Images were quantified using ImageJ software v1.52a (NIH, USA).

Antibodies:

LC3B (NB600-1384, Novus Biological), Sigma-Aldrich), Caspase-9 (9502, Cell Signalling), Cleaved Casapse-8 (9496, Cell Signalling), PERK (3192, Cell Signalling), Phospho-elF2α (3398, Cell Signalling) elF2α (5324, Cell Signalling), ATF-4 (11815, Cell Signalling), Phospho-Akt (4060, Cell Signalling), Akt (9272, Cell Signalling), Phospho-Atg14 (Ser29) (92340, Cell signalling), Phospho-PRAS40 (Thr246) (13175T, Cell signalling), mTOR (7C10) (2983, Cell Signalling), Phospho-mTOR (2971, Cell Signalling), and Phospho-myc Ser62 (13748S, Cell Signalling).

Colony formation assay

1x104 DLBCL cells/ 140µl well were seeded in replicates in a clear flat bottom 96-well plate and treated for 24 h, respectively. Cells were collected and resuspended in 1.5ml MethoCult H4230 (STEMCELL Technologies, USA) supplemented with 1% Penicillin and Streptomycin (Sigma-Aldrich). A monolayer was formed in 6-well plates. On day 8 images were taken using

a Leica microscope and software. Five independent images were taken per well and a mean was generated. A mean of the replicated wells was combined generating number of colonies (mean) per field of view.

High-throughput transcriptome analysis using next generation RNA sequencing

1x106 cells were cultured in 6-well plates and treated with 2.5µM MRT68921, or Vehicle for 1 h. Total RNA was extracted using a miRNeasy Tissue/ Cells Advanced Micro kit following the manufacturer's instructions (Qiagen, Germany). Total RNA was sent to Microsynth AG (Switzerland) for sequencing. Library was prepared using Illumina stranded, poly-A enriched, TruSeq RNA libraries. Sequencing was done using Illumina NextSeq, v2.5, 1x75bp (30 million single end reads per sample). Demultiplexing and trimming of Illumina adaptor residuals. Reads were mapped to human reference genome (GRCh38/hg38) and analysed after TMM (edgeR) normalisation and log2 transformation. Genes with mean values a log2 TMM>1 for at least one of the conditions were kept into the analysis. Two-way ANOVA was used to analyse the dataset. Gene expressions with significant differences at p-value1.5 were selected. Variations among the profiled samples were mapped using principal component analysis (PCA). Data clustering was established using Euclidean's method based on the average linkage. Heatmaps were generated according to the standard normal distribution of the values.

Gene Ontology and Gene Set Enrichment Analysis (GSEA)

For gene ontology (GO) enrichment, the list of differently expressed genes was grouped into functional hierarchies. Chi-square test was used to calculate the enrichment scores comparing the proportion of the gene list in a group to the proportion of the background genes/ proteins using GO resource database (geneontology.org), KEGG mapping (www.genome.jp/kegg) and Enrichr database (amp.pharm.mssm.edu/Enrichr). GO enrichment scores valued at 3 or above corresponded to significant differential expressed cellular process/ pathway ($p<0.05$). Gene set enrichment analysis (GSEA) was performed using GSEA software v.4 (Broad institute, Cambridge). Enrichment analysis was assessed for all genes relative to pathways obtained from Pathcards database (pathcards.genecards.org) or from the Broad Institute's Molecular Signatures Database (MsigDB).

Statistical analysis

GraphPad Prism v9.4 (GraphPad Software, USA) was used for data analysis. Analysis of variance (ANOVA), followed by post hoc Dunnett for multiple comparisons, or Student's *t*-test two tailed, or Mann-Whitney *U*-tests were applied to compare between study groups. Kaplan-Meier curve produced the time-to-event data and Log-rank (Mantel-Cox) test compared the probability of survival between differential gene expression. To calculate the overlap between two groups of genes we used http://nemates.org/MA/progs/overlap_stats.html. *P* values less than 0.05 were considered significant.

Method References

1. Zlobec I, Suter G, Perren A, Lugli A. A next-generation tissue microarray (ngTMA) protocol for biomarker studies. *J Vis Exp* 2014 Sep 23; (91)**:** 51893.

2. Schläfli AM, Berezowska S, Adams O, Langer R, Tschan MP. Reliable LC3 and p62 autophagy marker detection in formalin fixed paraffin embedded human tissue by immunohistochemistry. *Eur J Histochem* 2015 May 5; **59**(2)**:** 2481.

3. Davies A, Cummin TE, Barrans S, Maishman T, Mamot C, Novak U, *et al.* Gene-expression profiling of bortezomib added to standard chemoimmunotherapy for diffuse large B-cell lymphoma (REMoDL-B): an open-label, randomised, phase 3 trial. *Lancet Oncol* 2019 May; **20**(5)**:** 649-662.

4. Camp RL, Dolled-Filhart M, Rimm DL. X-tile: a new bio-informatics tool for biomarker assessment and outcome-based cut-point optimization. *Clin Cancer Res* 2004 Nov 1; **10**(21)**:** 7252-7259.
