# Supplementary material for: Analysis of autophagy in DLBCL reveals subtype-specific differences and the preferential targeting of ULK1 inhibition in GCB-DLBCL provides a rationale as a new therapeutic approach
Source: Leukemia. 2024 Jan 23;38(2):424–9. doi: 10.1038/s41375-024-02147-4 (PMC10844068; doi:10.1038/s41375-024-02147-4)
Supplement: Supplementary file 2 — Supplementary Figures [file 41375_2024_2147_MOESM2_ESM.pdf]

A)

Subtype	Cytoplasmic staining (no. of cases)			
	LC3B (n=)		p62 (n=)	
	Low	High	Low	High
DLBCL, NOS (n=100)	41	59	42	58
FL (n=37)	13	24	25	12

B)

	Lymphoma Subtypes	Total no. of cases	No. of LC3B cases		Percentage of LC3B staining (%)	
			Low	High	Low	High
Indolent	CLL/SLL	37	26	11	70.3	29.7
	FL	37	13	24	35.1	64.9
	MZL	28	25	3	89.3	10.7
	MCL	47	36	11	76.6	23.4
Aggressive	BL	16	5	11	31.3	68.8
	DLBCL, CNS	36	2	34	5.6	94.4
	DLBCL, NOS	100	41	59	41	59
	PMBL	9	4	5	44.4	55.6
	HGB	8	2	6	25	75

C)

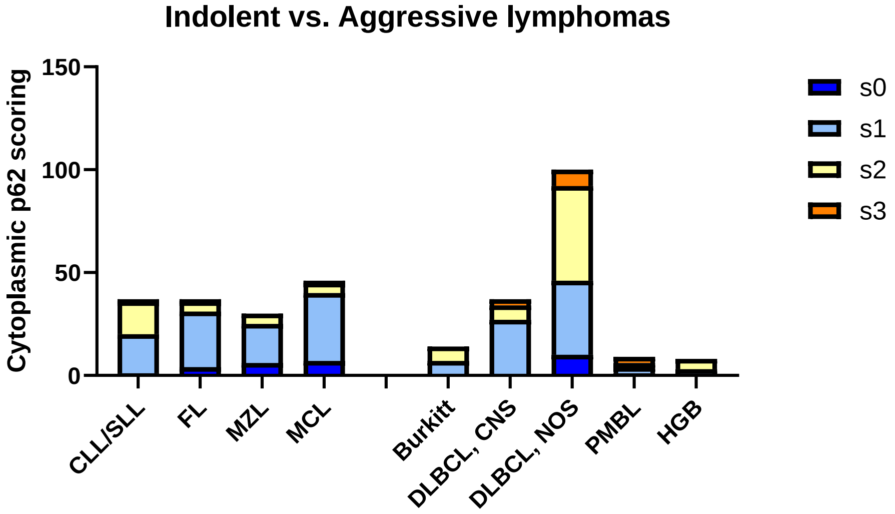

D)

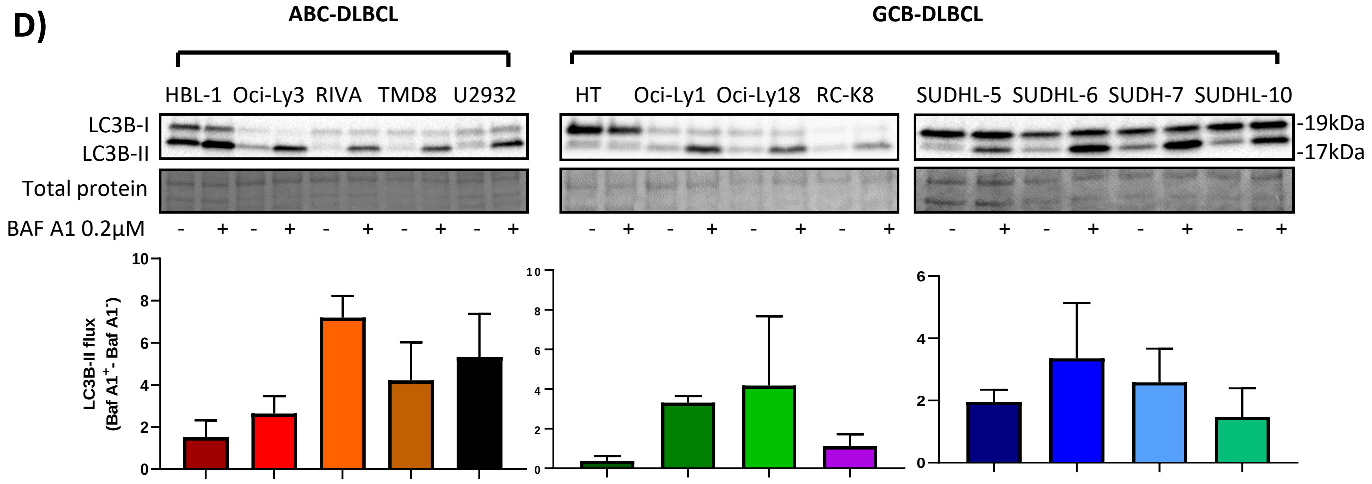

E)

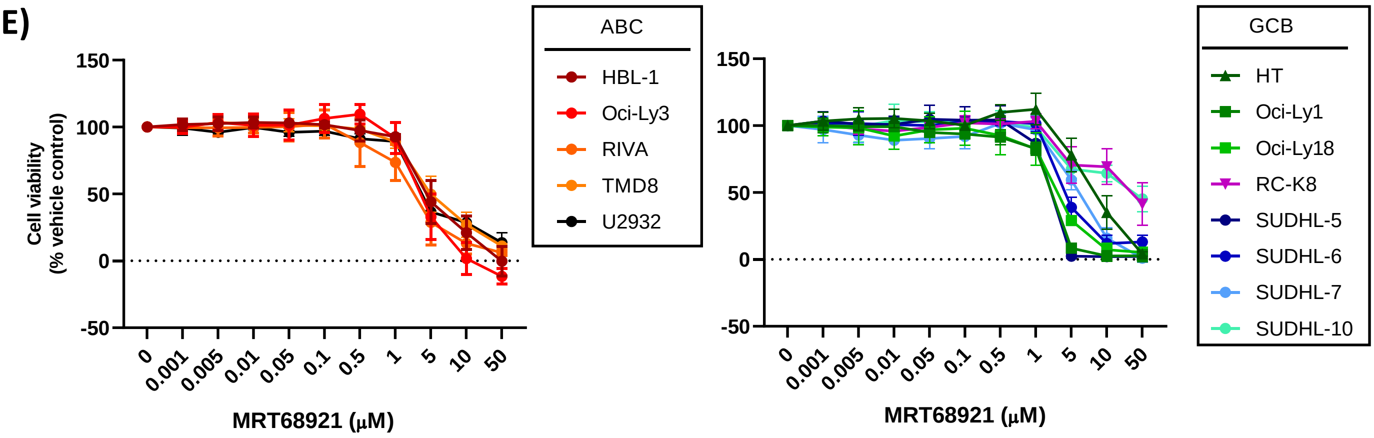

Supplementary Figure 1

Supplementary figure 1: Primary aggressive lymphomas exhibit increased LC3B cytoplasmic staining and DLBCL subtype cell lines exhibit deregulated autophagy. **A)** Total number of DLBCL, NOS and FL cases (no. of cases) staining positive for LC3B and p62 were tabled. **B)** 318 primary lymphoma cases were grouped according to their clinical classifications being indolent (n=149) and aggressive (n=169) lymphomas. Total number of B-cell lymphoma cases, LC3B high and low cases, and percentage of cytoplasmic LC3B staining was tabled. **C)** p62 staining on indolent and aggressive primary lymphoma biopsies. Scoring system of p62 staining intensity 0-3, score 0 lack of immunoreactivity, score 1 weak staining, score 2 intermediate dot-like staining and score 3 strong immunoreactivity in in ≥ 5% of the cells. **D)** ABC and GCB cell lines were treated with Bafilomycin A1 (Baf A1, 0.2μM) for 2 h. Image J was used to quantify the bands and normalized to control- treated cells (BafA1^{+}-BafA^{-}). Total protein was used as a loading control. Representative blot from at least three independent experiments is shown. Plotted bar graphs represent mean ± standard deviation (SD). **E)** DLBCL cell lines were treated with MRT68921 (at indicated concentrations) for 24 h. Cell viability was measured using PrestoBlue assay. Values were normalised to untreated (four independent experiments, mean± SD).

Indolent Lymphoma: CLL: chronic lymphocytic leukaemia; SLL: small lymphocytic lymphoma; FL: follicular lymphoma; MZL: marginal zone lymphoma; MCL: mantle cell lymphoma; Aggressive lymphoma: BL: burkitt lymphoma; DLBCL, CNS: diffuse large B-cell lymphoma, central nervous system; DLBCL, NOS: diffuse large B-cell lymphoma, not otherwise specified; PMBL: primary mediastinal B-cell lymphoma; HGB: high-grade B-cell lymphoma.

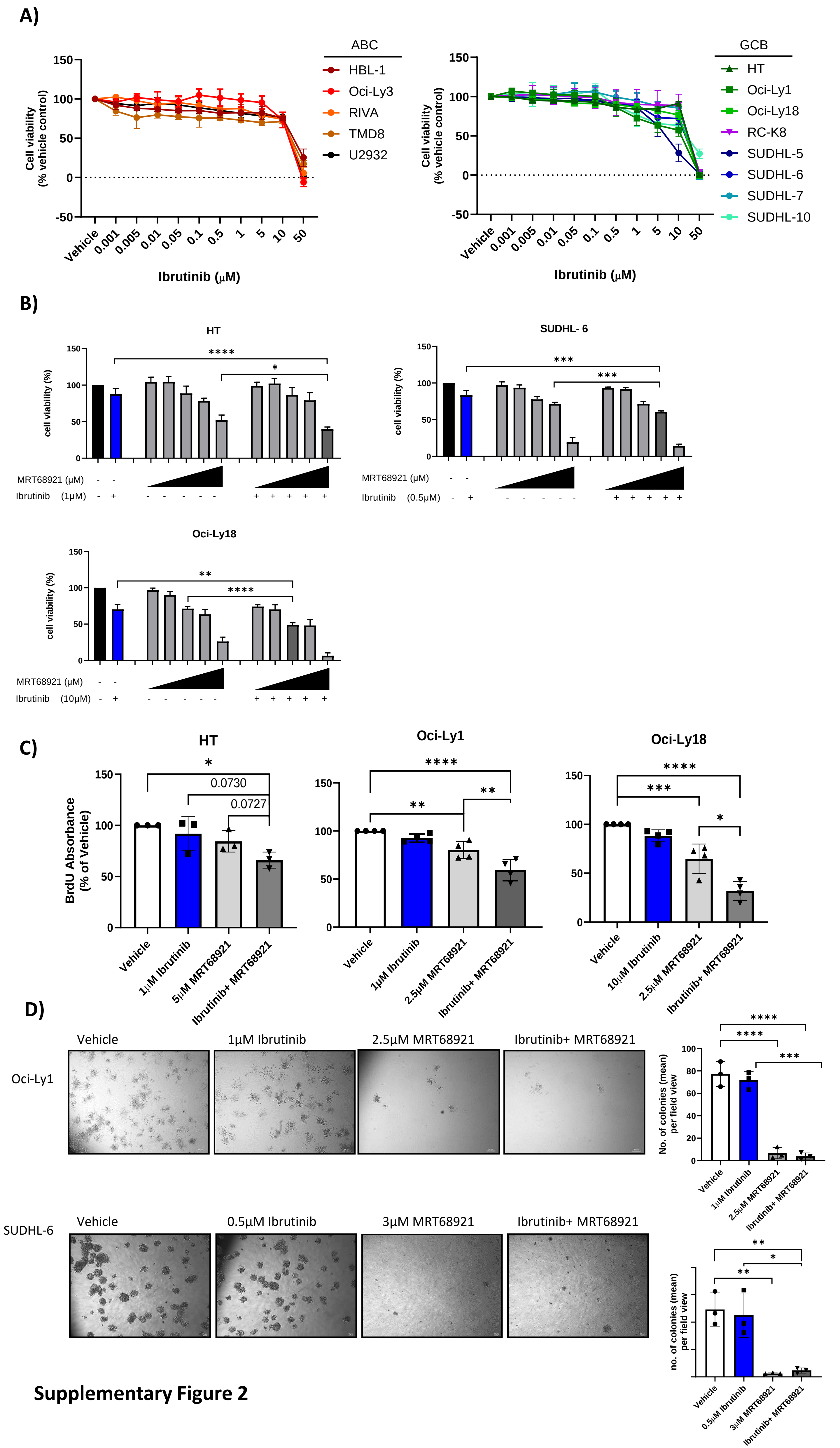

Supplementary Figure 2

Supplementary figure 2: Ibrutinib and MRT68921 cooperatively elicits anti-tumour activity *in vitro* in GCB-DLBCL cell lines. **A)** ABC and GCB cell lines were dosed with different concentrations of ibrutinib for 24 h. Cell viability using PrestoBlue was assessed (four independent experiments, error bars represent mean+ SD). **B)** GCB HT, SUDHL-6 and Oci-Ly18 cell lines were treated with vehicle, ibrutinib, ascending MRT68921 (0.5, 1, 2.5, 3 and 5 μM) and in combination (concentrations as indicated) for 24 h, cellular viability was evaluated. **C)** To evaluate cell proliferation the incorporation of BrdU was measured in HT, Oci-Ly1 and Oci-Ly18 treated with ibrutinib ± MRT68921 for 24hr. The SUDHL-6 cell line was not suitable for this assay. *P* values were calculated using one-way ANOVA and Dunnett's multiple comparisons test, and student *t* test between variables. The data is representative of at least three independent experiments and error bars of mean± SD **D)** GCB cell lines were treated with ibrutinib ± MRT68321 as indicated for 24 h. Cells were then collected and seeded in six well plates containing methylcellulose and incubated for 8 days. One-way ANOVA with post hoc Dunnett's multiple comparisons test was applied and Student *t* test unpaired two tailed test. The data is representative of at least three independent experiments and error bars of mean± SD, $*P<0.05$, $**P<0.01$, $***P<0.001$, $****P<0.0001$.

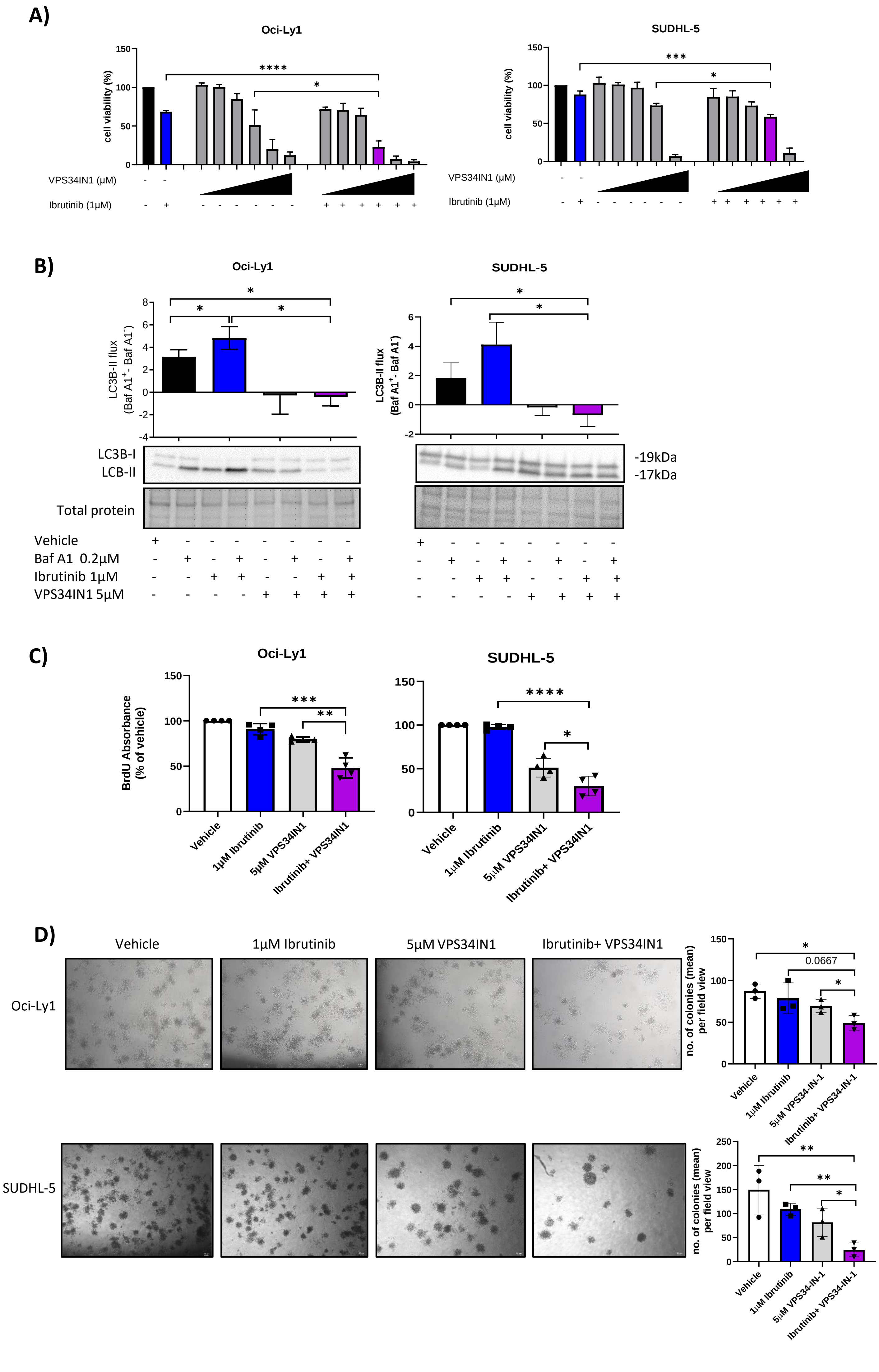

Supplementary Figure 3

Supplementary figure 3: VPS34 inhibition reduces autophagic flux and targets colony formation. **A)** To assess whether inhibition of the secondary autophagy initiation complex: VPS34 may sensitise the GCB Oci-Ly1 and SUDHL-5 cell lines to ibrutinib we titrated different concentrations of VPS34 inhibitor (VPS34IN1) (0.5, 1, 2.5, 5, 10 µM) in the presence and absence of ibrutinib (1µM) and vehicle for 24 h. Cell viability was measured using PrestoBlue in four independent experiments. *P* values were calculated using one-way ANOVA and Dunnett's multiple comparisons test, and student t test between variables. **B)** Autophagic flux was measured. Quantification of LC3B turnover was determined using Mann-Whitney U test in four independent experiments. **C)** Effects of ibrutinib + VPS34IN1 24 h on proliferation was assessed using BrdU assay. Co-treatment of ibrutinib and VPS34IN1 significantly reduced cellular proliferation. **D)** Using the selected concentrations (as indicated) colony forming capacity was assessed. One-way ANOVA with post hoc Dunnett's multiple comparisons test was applied and also Student t test unpaired two tailed test. The data is representative of three independent experiments. Error bars correspond to the mean $\pm$ SD, *$P<0.05$, **$P<0.01$, ***$P<0.001$, ****$P<0.0001$

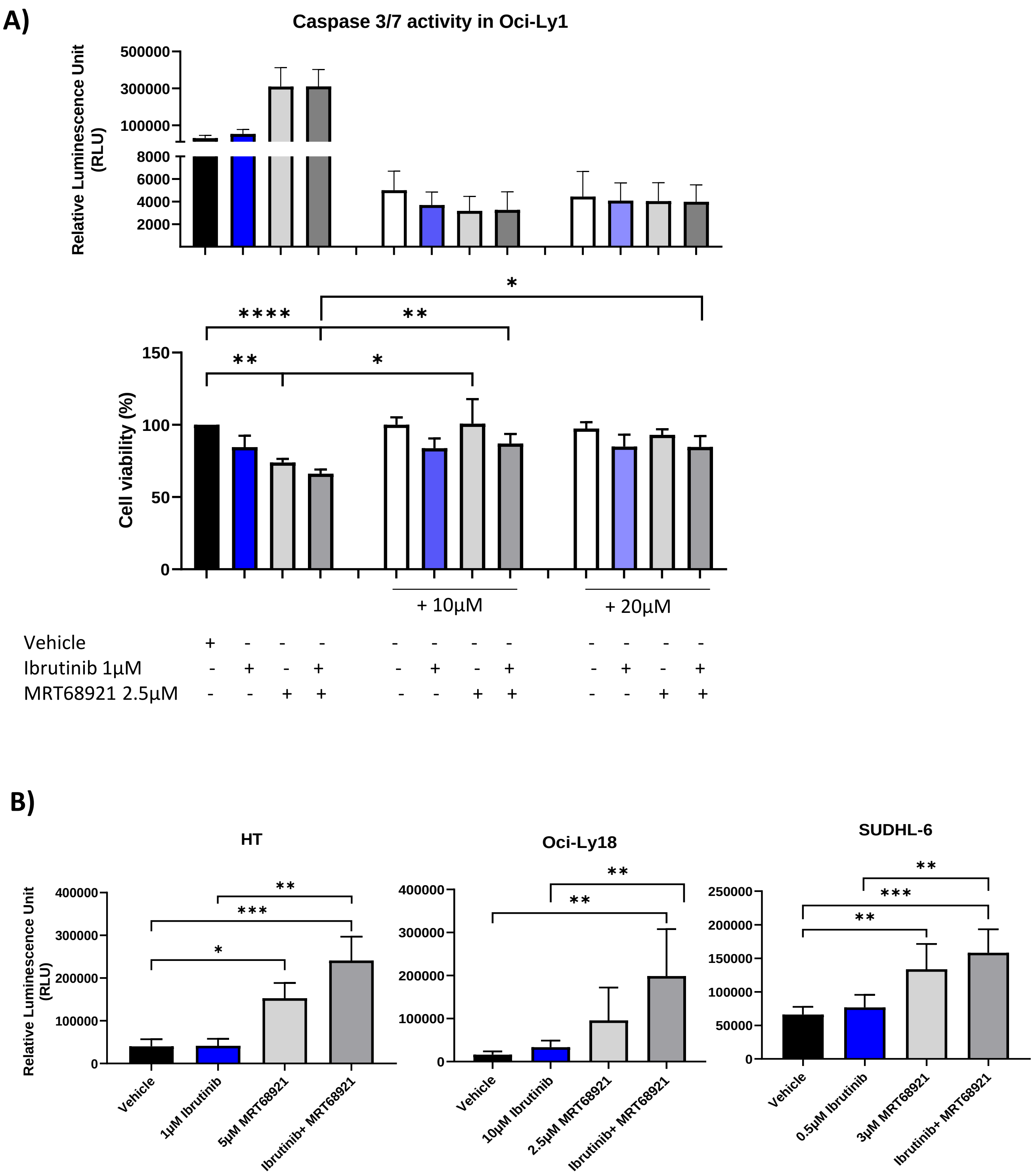

Supplementary Figure 4

Supplementary figure 4: Pan-caspase inhibitor Q-VD-OPh blocked effector caspases 3/7 activity and rescued GCB Oci-Ly1 cells from apoptosis. **A)** GCB Oci-Ly1 cells were pretreated with either 10 or 20µM for 1 h thereafter vehicle, 1µM ibrutinib, 2.5µM MRT68921 and combination was added. Caspase 3/7 activity was measured using luminescence and cell viability was measured at 8 h (three independent experiments were conducted). **B)** GCB HT, Oci-Ly18 and SUDHL-6 cell lines were treated as indicated for 8 h. Caspase 3/7 activity was evaluated. Error bars show the mean ± SD, *P<0.05, **P<0.01, ***P<0.001, ****P<0.0001

A)

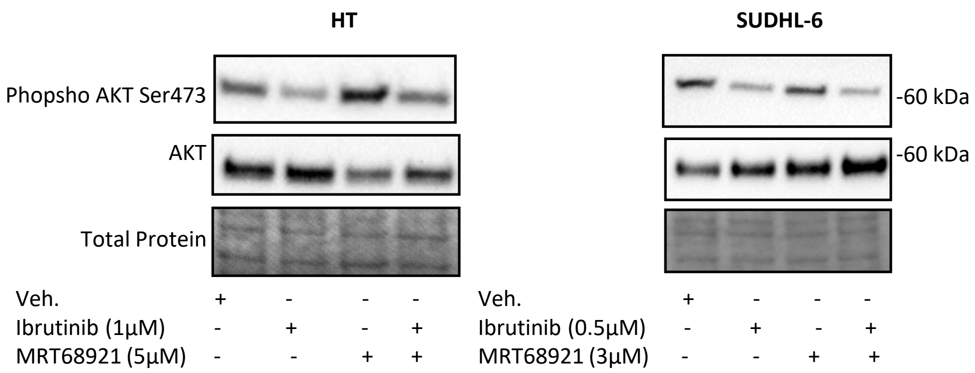

B)

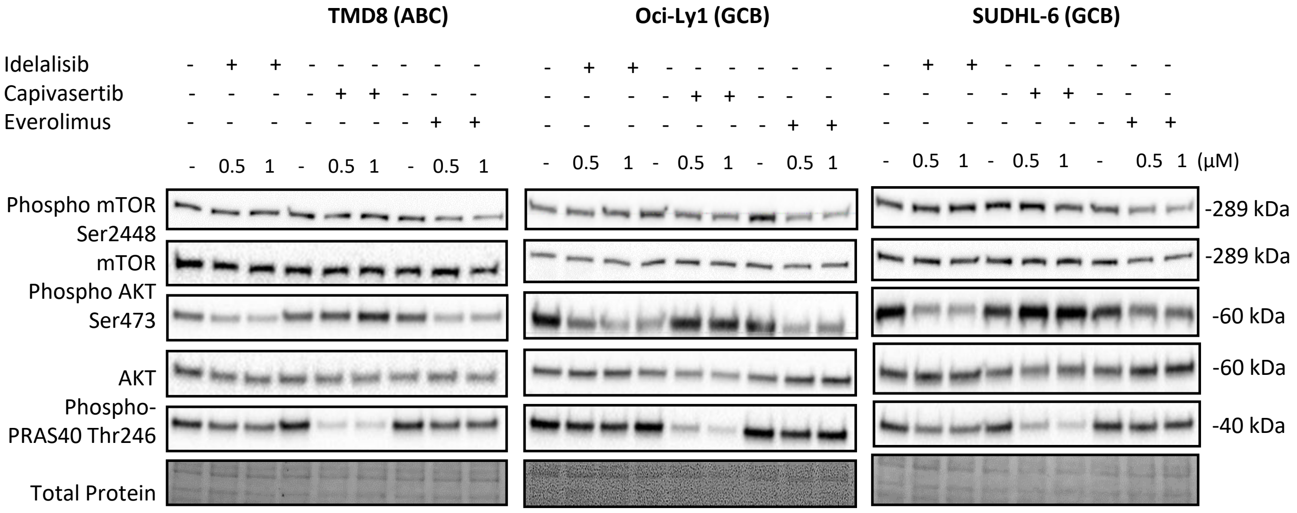

Supplementary Figure 5

Supplementary figure 5: Immunoblotting to assess the phosphorylation of AKT Ser473 and total AKT. Cell lines were treated with vehicle, ibrutinib, MRT68921 and co-treatment (as indicated) for 24 h. Representative blot of three independent experiments. To validate ibrutinib targeted the AKT pathway ABC TMD8 and GCB Oci-Ly1 and SUDHL-6 cell lines were treated with idelalisib (p110δ isoform PI3K inhibitor), capivasertib (pan-AKT kinase ATP-competitive inhibitor) and everolimus (mTORC1 inhibitor) for 4 h (concentrations as indicated). Representative blot indicate that pharmacological inhibitors targeting the PI3K-AKT-mTOR decreased the phosphorylation of active proteins associated to the pathway therefore confirming that ibrutinib is active in dephosphorylating AKT.

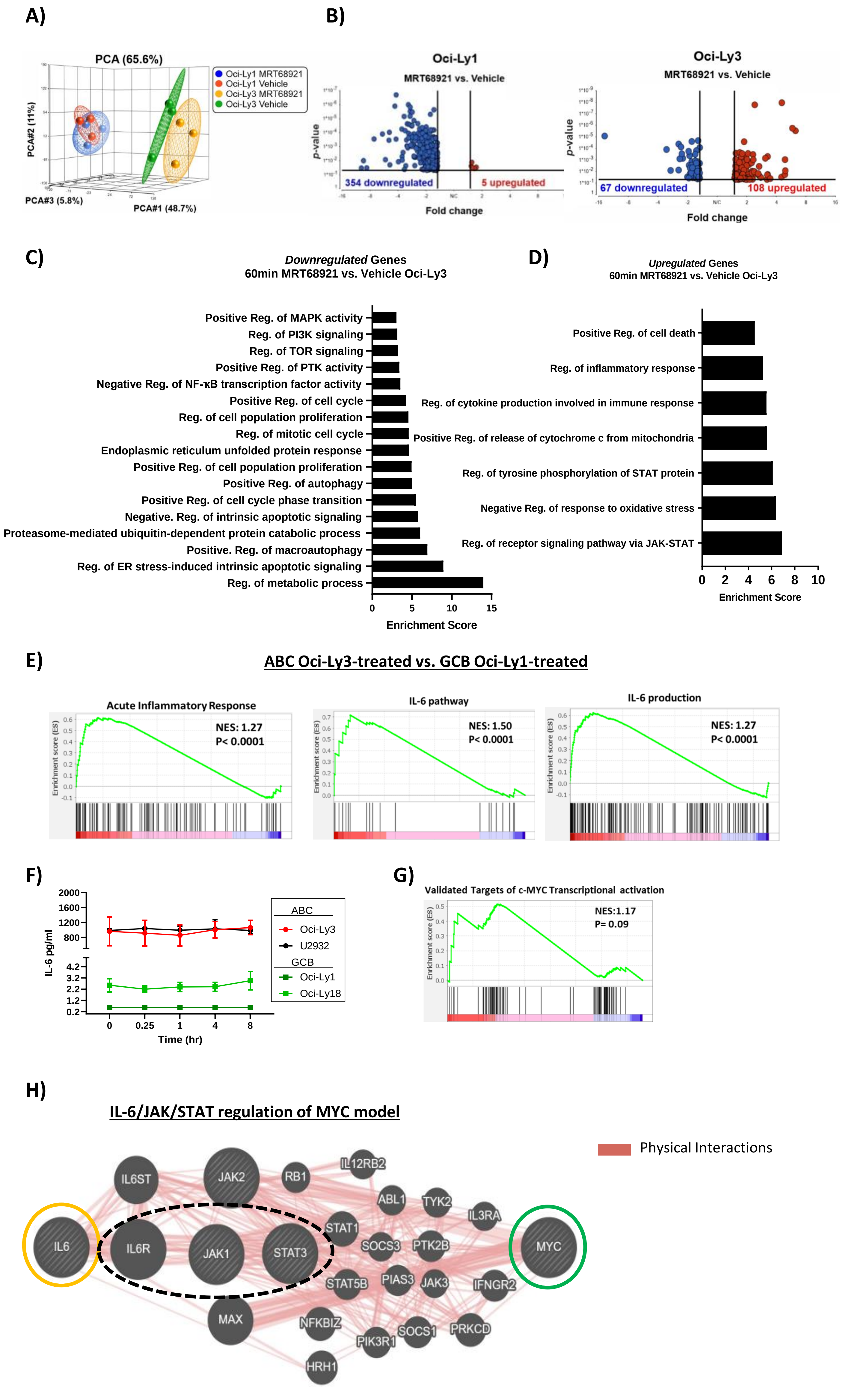

Supplementary Figure 6

Supplementary figure 6: Distinct ULK1 inhibitory pathways in DLBCL subtype cell lines. **A)** Principle component analysis (PCA) mapped to demonstrate the variability of GCB Oci-Ly1 and ABC Oci-Ly3 treated with MRT68921 2.5µM and vehicle for 1 h (three independent experiments). **B)** Volcano plots illustrates the differentially expressed genes of GCB Oci-Ly1 and ABC Oci-Ly3 MRT68921-treated vs. vehicle; *y* axis: negative log of *P* value, *x* axis: log2-fold change; blue dots represent downregulated genes and red dots upregulated genes. **C)** Gene Ontology (GO) analysis of downregulated genes in ABC Oci-Ly3 (MRT68921-treated vs. vehicle). **D)** GO enrichment of differentially expressed genes upregulated in ABC Oci-Ly3 (MRT68921-treated vs. vehicle). **E)** Gene set enrichment analysis (GSEA) reports distribution of differentially expressed genes through normalised enrichment score (NES) of ABC Oci-Ly3-MRT68921 treated vs. GCB Oci-Ly1-MRT68921. **F)** IL-6 secretion remained significantly higher in ABC cell lines, in contrast to GCB cell lines. ULK1 inhibition did not change the secretion of IL-6 in a time-dependent manner (h). Data shown is a relative cytokine unit (pg/ml), four independent experiments was conducted in Oci-Ly3, Oci-Ly1 and Oci-Ly18 cell lines, U2932 was independently repeated twice. Error bars represent the mean $\pm$ SD, ****P<0.0001). **G)** GSEA demonstrating no significant changes in c-MYC transcriptional transcripts in Oci-Ly3 (MRT68921-treated vs. vehicle). **H)** *In silico* assessment of predicted protein-protein interactions between IL-6, JAK1, STAT3 and MYC using GeneMANIA database (http://genemania.org/).

A)

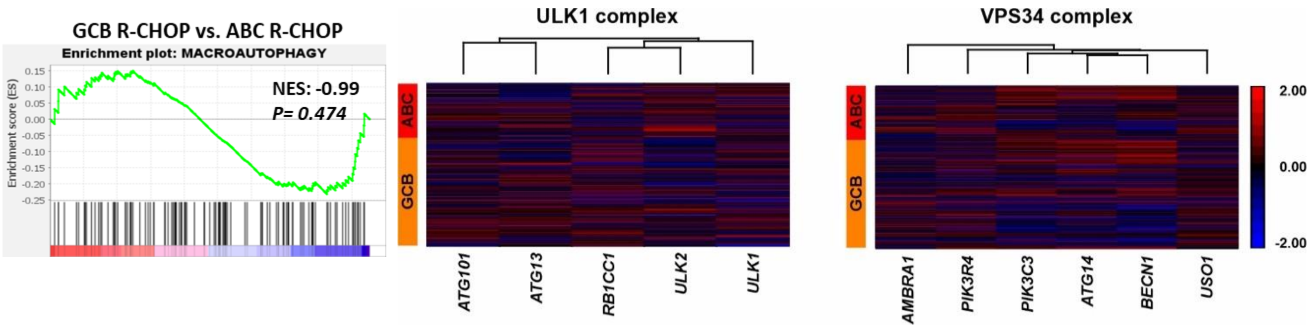

B)

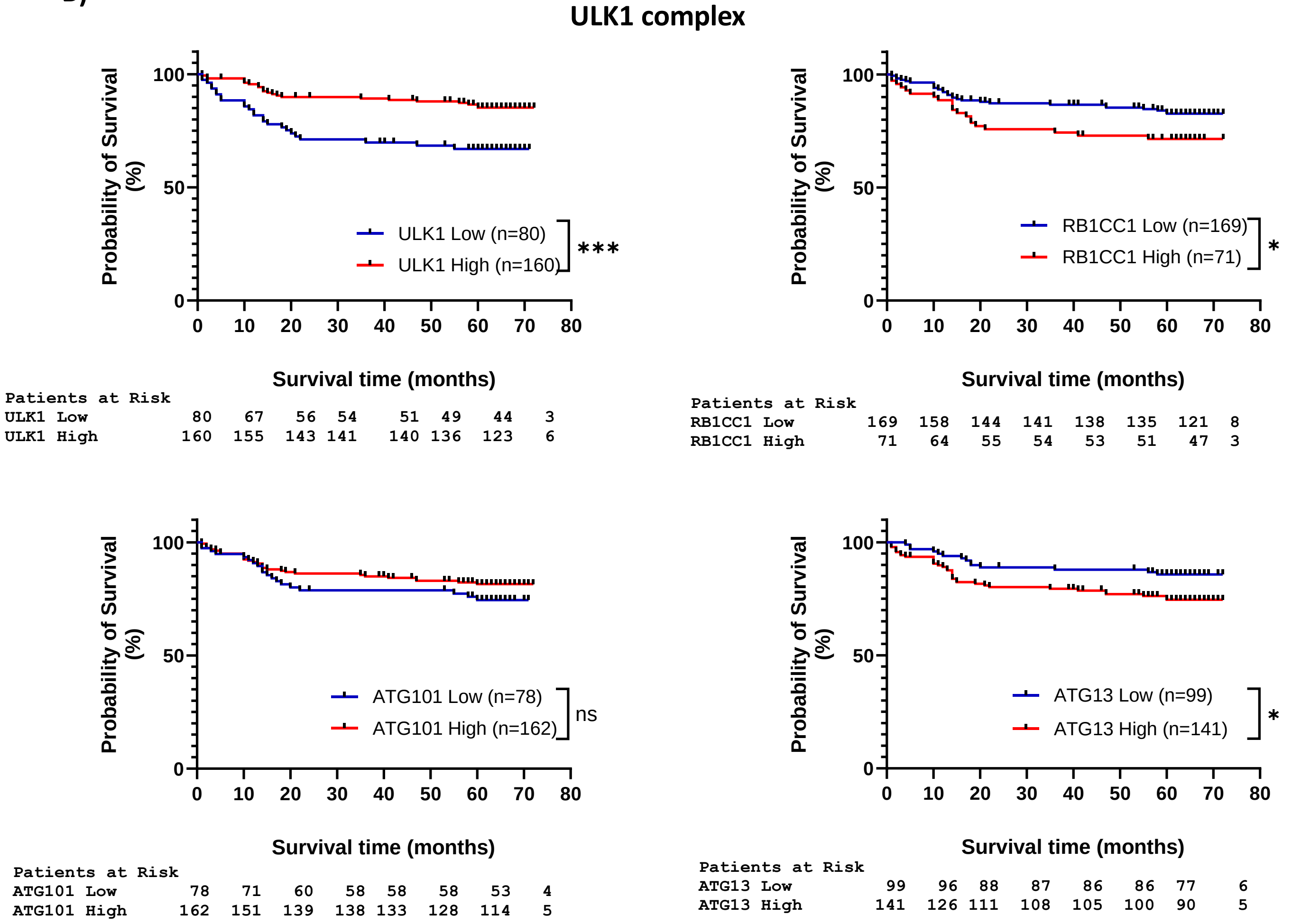

Supplementary Figure 7

Supplementary figure 7: Autophagy initiating *ATGs* expression in GCB patients and survival outcome relating to R-CHOP. **A)** GSEA compared autophagy transcripts between gene expression profiling (GEP) of GCB (n=240) and ABC (n=121) patients assigned to R-CHOP treatment arm[1]. Heat-maps of differentially expressed genes within ULK1 complex and VPS34 complex in ABC and GCB patients. **B)** Autophagy initiating ATGs expression in GCB patients and survival outcome relating to R-CHOP. Cut-off points for differentially expressed genes of the ULK1 complex was determined using X-tile software[2]. Kaplan-Meier analysis of and log-rank test (mantel-cox) provided statistical significance between gene expression.

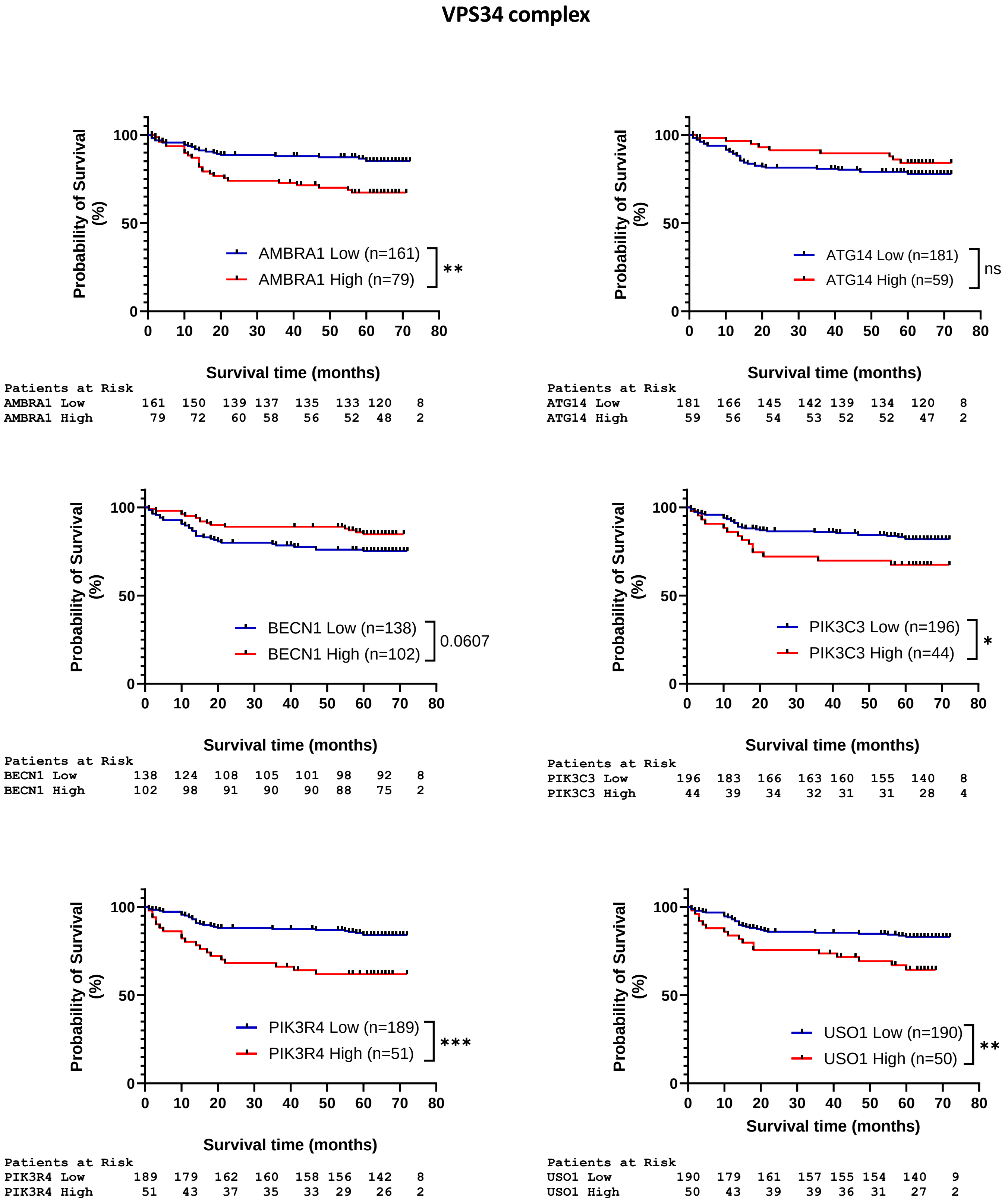

Supplementary Figure 8

Supplementary figure 8: VPS34 gene expression in GCB patients and survival outcome relating to R-CHOP. Gene expression profiles of GCB patients (n=240)[1], as mentioned (supplementary Figure 7B). Cut-off points for differentially expressed genes from the VPS34 complex was determined using X-tile software[2]. Kaplan-Meier analysis of and log-rank test (mantel-cox) provided statistical significance between gene expression for survival outcome.

Autophagy initiating complex	Gene comparison	Gene 1	Gene 2	Gene overlap	p value	*Transformed* p value (-Log10)
**ULK1 complex**	ULK1 vs. ATG13	160	141	96	1.7E-175	174.8
	ATG13 vs. RB1CC1	141	71	32	2.3E-49	48.6
	RB1CC1 vs. ULK1	71	160	46	8.2E-79	78.1
**VPS34 complex**	PIK3C3 vs. AMBRA1	44	79	24	1.5E-46	45.8
	AMBRA1 vs. USO1	79	50	16	2.3E-26	25.6
	USO1 vs. PIK3R4	50	51	7	1.0E-10	10.0
	PIK3R4 vs. PIK3R4	44	51	20	3.9E-41	40.4
	PIK3C3 vs. USO1	44	50	8	3.8E-13	12.4
	AMBRA1 vs. PIK3R4	79	51	29	3.3E-57	56.5

Supplementary Figure 9

Supplementary figure 9: ULK1 complex is dominantly expressed in GCB patients and correlates with inferior survival outcome relating to R-CHOP treatment. **A)** Probability of identifying overlapping genes was calculated by comparing two independent genes and within the intersection. Statistical analysis was calculated using http://nemates.org/MA/progs/overlap_stats.html.

Supplementary References

1. Davies A, Cummin TE, Barrans S, Maishman T, Mamot C, Novak U, *et al.* Gene-expression profiling of bortezomib added to standard chemoimmunotherapy for diffuse large B-cell lymphoma (REMoDL-B): an open-label, randomised, phase 3 trial. *Lancet Oncol* 2019 May; **20**(5)**:** 649-662.

2. Camp RL, Dolled-Filhart M, Rimm DL. X-tile: a new bio-informatics tool for biomarker assessment and outcome-based cut-point optimization. *Clin Cancer Res* 2004 Nov 1; **10**(21)**:** 7252-7259.
